# Chronic Disease Disparities by County Economic Status and Metropolitan Classification, Behavioral Risk Factor Surveillance System, 2013

**DOI:** 10.5888/pcd13.160088

**Published:** 2016-09-01

**Authors:** Kate M. Shaw, Kristina A. Theis, Shannon Self-Brown, Douglas W. Roblin, Lawrence Barker

**Affiliations:** Author Affiliations: Kristina A. Theis, Lawrence Barker, Centers for Disease Control and Prevention, Atlanta, Georgia; Shannon Self-Brown, Douglas Roblin, Georgia State University School of Public Health, Atlanta, Georgia. Dr Shaw is also affiliated with Georgia State University School of Public Health, Atlanta, Georgia.

## Abstract

**Introduction:**

Racial/ethnic disparities have been studied extensively. However, the combined influence of geographic location and economic status on specific health outcomes is less well studied. This study’s objective was to examine 1) the disparity in chronic disease prevalence in the United States by county economic status and metropolitan classification and 2) the social gradient by economic status. The association of hypertension, arthritis, and poor health with county economic status was also explored.

**Methods:**

We used 2013 Behavioral Risk Factor Surveillance System data. County economic status was categorized by using data on unemployment, poverty, and per capita market income. While controlling for sociodemographics and other covariates, we used multivariable logistic regression to evaluate the relationship between economic status and hypertension, arthritis, and self-rated health.

**Results:**

Prevalence of hypertension, arthritis, and poor health in the poorest counties was 9%, 13%, and 15% higher, respectively, than in the most affluent counties. After we controlled for covariates, poor counties still had a higher prevalence of the studied conditions.

**Conclusion:**

We found that residents of poor counties had a higher prevalence of poor health outcomes than affluent counties, even after we controlled for known risk factors. Further, the prevalence of poor health outcomes decreased as county economics improved. Findings suggest that poor counties would benefit from targeted public health interventions, better access to health care services, and improved food and built environments.

## Introduction

Chronic diseases affect 117 million American adults, with almost 60 million having more than one chronic condition (1). However, the burden of chronic disease is not shared equally. Although racial/ethnic disparities are well known, the combined influence of geographic and economic status on specific health outcomes is less well studied (2). A close examination of the relationship between economic indicators and health conditions might provide important information and insight into the intervention type best suited for particular groups.

Despite many advances in public health (3), much remains to be done in prevention and control of morbidity and mortality, particularly chronic conditions (2). As demonstrated in countries at all income levels, poor health outcomes increase as socioeconomic position decreases (referred to as the “social gradient”: ie, inequalities in health status are related to inequalities in social status) (4).

Socioeconomics can be measured by using individual characteristics (eg, education or household income) or community characteristics (eg, percentage of residents living in poverty). Social determinants can drive gradient-related health outcomes, and these can be further categorized as downstream (more proximal to the individual) or upstream (more distal from the individual) (5). The most distal social determinants are socioeconomic opportunities and resources, which affect living and working environments. The most downstream determinants that directly affect an individual’s health are behaviors and level of access to medical care.

In the United States, economic opportunities and resources, as measured by an area’s poverty level, are associated with mortality. Galea and colleagues estimated that more than 39,000 deaths in 2000 were attributed to area-level poverty (6).

Here, we examine 1) the prevalence of selected chronic diseases by US county economic status and by metropolitan classification and 2) the social gradient by county economic status, both overall and by metropolitan classification. We use multivariable logistic regression to examine the association between county economic status and prevalence of hypertension, arthritis, and poor health, after controlling for risk factors.

## Methods

Data from the 2013 Behavioral Risk Factor Surveillance System (BRFSS) were analyzed. The BRFSS is a random-digit–dialed survey of noninstitutionalized civilian adults 18 years or older in the United States (7). To account for probability of selection and population distribution, the BRFSS uses a complex sample survey design and weighting. Survey data from both landline and cell‑phone–only households were used.

Respondents were asked questions related to their health, risky behaviors, and use of health services. They were asked whether a health professional ever told them they have specific chronic diseases and whether they have self-reported risk factors. We examined data on leisure-time physical activity, poor health, body mass index classified as overweight or obese, hypertension, high cholesterol, heart disease, arthritis, diabetes, current cigarette smoking, depressive disorder, asthma, and chronic obstructive pulmonary disease. Respondents were also asked to rate their general health as excellent, very good, good, fair, or poor; they were considered to have poor health if they answered that their health was fair or poor. We considered data as missing if respondents said that they did not know whether they had the condition, were not sure whether they had the condition, or refused to answer the question about the condition; we excluded missing data from analyses. The full survey questionnaire is available (8).

To measure county economic status, we used an index similar to the one the Appalachian Regional Commission (ARC) used for that purpose for many years. This method uses an index measure to assess economic distress in Appalachian counties (9). Three economic measures were used to create county economic status: 2013 unemployment rate, 2013 per capita market income, and 5-year (2009–2013) poverty rates. Unemployment estimates are provided by the US Bureau of Labor Statistics (10), data on per capita market income are from the US Bureau of Economic Analysis (11), and data on poverty level are from the US Census Bureau’s American Community Survey (12). For simplicity, we used only 1 year of unemployment data instead of the 3 years used by ARC; adding 2 additional years of data had little impact. An index variable was created by adding together the standardized values of each of these economic measures. The negative standardized values for unemployment and poverty were used to make smaller values more desirable. The summed values were categorized as quintiles. These quintiles were labeled poorest, poor, median, affluent, and most affluent.

County metropolitan classification was determined by using the US Office of Management and Budget’s metropolitan classifications: regions with at least 1 urbanized area of 50,000 or greater population and their socially and economically integrated adjacent areas were considered to be metropolitan (13). Because of data limitations, independent cities in Virginia (ie, cities not in the territory of any county) with populations of less than 100,000 were combined with their adjacent county; 10 independent cities had 100,000 or greater population and had economic measures available, so they were included as separate geographic regions in our analyses. Because Alaska and Hawaii have unique economies, analyses were limited to the contiguous United States, resulting in a total of 3,070 counties and 10 independent cities in Virginia (3,080 geographic regions in all). For convenience, we refer to all geographic regions (counties and independent cities) as “counties.” We also determined the economic status of each county and compared the metropolitan counties (1,939; 63.0%) with the nonmetropolitan counties (1,141; 37.0%). 

Several risk factors are associated with chronic disease, including not having health insurance, being obese, using tobacco, and not participating in physical activity (14). Participants were considered to have health insurance if they responded that they had any health care coverage at the time of the survey. Overweight or obese was defined as having self-reported height and weight measurements equivalent to a body mass index of 25.0 kg/m^2^ or higher. Respondents who smoked at least 100 cigarettes in their lifetime and stated that they still smoke were considered current smokers. Respondents were considered physically active if they participated in any leisure-time physical activities during the previous month.

County of residence was not available for 22,639 (4.8%) respondents, so data on those respondents were excluded. The final analytic sample consisted of 448,790 respondents in 3,064 counties and 10 Virginia cities (3,074 counties). Analyses were conducted using SAS (version 9.3) callable SUDAAN, release 11.0.1 (15), to account for the complex survey design. We used multivariable logistic regression to examine the relationship between county economic status and hypertension, arthritis, and self-rated health status after controlling for sociodemographic and health risk factors (age, sex, race/ethnicity, education, household income, inadequate or no health insurance, overweight or obesity, current cigarette smoking, lack of physical activity), and metropolitan county classification. Weighted percentages, adjusted prevalence ratios, and their 95% confidence intervals were calculated. To assess disparities among economic groups, the estimate for the group of poorest counties was subtracted from that for the most affluent group of counties. Confidence intervals for the differences that do not include zero indicate a significant difference at the 2-sided α of .05. Estimates were calculated overall and for metropolitan and nonmetropolitan counties separately. Adjusted prevalence ratios were calculated using SUDAAN’s logistic procedure and the predicted marginal statement with the adjusted risk ratios option (15).

## Results

Demographic characteristics differed by metropolitan classification (Table 1). Most respondents lived in metropolitan counties (weighted percentage, 83.5%). Compared with nonmetropolitan residents, those living in metropolitan counties were more likely to be aged 18 to 44 years, Hispanic or non-Hispanic black, and a college graduate; they were also more likely to have household incomes of $75,000 or higher. In contrast, nonmetropolitan respondents were more likely to be aged 65 years or older, be non-Hispanic white, have less than a college education, and have a household income of less than $75,000.

**Table 1:** Prevalence of Chronic Disease and Risk Factors by County Metropolitan Classification^a^ and Differences Between Most Affluent and Poorest Counties^b^, Adults (≥18 y), Behavioral Risk Factor Surveillance System, 2013^c^

Characteristic	Overall, % (95% CI)^d^	Overall Difference Between Most Affluent and Poorest, Percentage Points (95% CI)^d^	Metro, % (95% CI)^d^	Metro Difference Between Most Affluent and Poorest, Percentage Points (95% CI)^d^	Nonmetro, % (95% CI)^d^	Nonmetro Difference Between Most Affluent and Poorest, Percentage Points (95% CI)^d^
**No. of respondents (%)**	448,790^e^ (100) **d**	—	306,648 **d** (83.5)^d^	—	142,142 **e** (16.5)^d^	—
**Age, y**						
18–44	44.7 (44.4 to 45.1)	−0.8 (−1.9 to 0.3)	45.5 (45.2 to 45.9)	−1.5 (−2.6 to −0.4)	40.8 (40.2 to 41.4)	−2.4 (−4.1 to −0.6)
45–64	35.6 (35.3 to 35.9)	1.7 (0.7 to 2.7)	35.5 (35.2 to 35.8)	2.2 (1.1 to 3.2)	36.3 (35.8 to 36.8)	1.4 (−0.2 to 2.9)
≥65	19.6 (19.4 to 19.8)	−0.9 (−1.6 to −0.2)	19.0 (18.8 to 19.2)	−0.7 (−1.4 to 0.1)	22.9 (22.5 to 23.3)	1.0 (−0.2 to 2.2)
**Sex**						
Male	48.2 (47.9 to 48.5)	1.1 (−0.1 to 2.2)	48.0 (47.7 to 48.4)	1.0 (−0.1 to 2.2)	48.8 (48.3 to 49.4)	2.9 (1.2 to 4.6)
Female	51.8 (51.5 to 52.2)	−1.1 (−2.2 to 0.1)	52.0 (51.6 to 52.3)	−1.0 (−2.2 to 0.1)	51.2 (50.6 to 51.8)	−2.9 (−4.6 to −1.2)
**Race/ethnicity**						
Hispanic	15.1 (14.9 to 15.4)	−8.1 (−9.2 to −7.1)	16.8 (16.5 to 17.2)	−12.2 (−13.2 to −11.1)	6.6 (6.2 to 6.9)	−0.6 (−1.9 to 0.7)
White, non-Hispanic	66.1 (65.7 to 66.4)	15.1 (14.0 to 16.3)	63.0 (62.6 to 63.3)	18.2 (17.0 to 19.3)	81.6 (81.1 to 82.1)	20.8 (19.1 to 22.5)
Black, non-Hispanic	11.9 (11.7 to 12.1)	−11.4 (−12.3 to −10.6)	12.7 (12.5 to 13.0)	−10.6 (−11.4 to −9.7)	7.9 (7.5 to 8.2)	−18.1 (−19.3 to −17.0)
Other, non-Hispanic **f**	6.9 (6.7 to 7.1)	4.4 (3.7 to 5.2)	7.5 (7.2 to 7.7)	4.6 (3.8 to 5.4)	3.9 (3.7 to 4.2)	−2.0 (−2.8 to −1.3)
**Education^g^**						
<High school graduate	15.2 (14.9 to 15.5)	−14.3 (−15.4 to −13.2)	14.8 (14.5 to 15.2)	−11.9 (−13.0 to −10.9)	17.2 (16.7 to 17.7)	−12.3 (−13.9 to −10.8)
High school graduate	27.9 (27.7 to 28.2)	−9.0 (−10.0 to −8.0)	26.3 (26.0 to 26.6)	−6.9 (−7.9 to −5.9)	36.2 (35.7 to 36.7)	−4.1 (−5.7 to −2.5)
Some college	29.8 (29.5 to 30.1)	0.8 (−0.2 to 1.9)	29.8 (29.5 to 30.2)	−1.5 (−2.5 to −0.4)	29.5 (29.0 to 30.0)	5.5 (3.9 to 7.0)
College graduate	27.1 (26.8 to 27.3)	22.5 (21.7 to 23.3)	29.1 (28.8 to 29.4)	20.3 (19.4 to 21.1)	17.1 (16.8 to 17.5)	10.9 (9.9 to 11.9)
**Annual household income, $**						
<25,000	31.0 (30.7 to 31.3)	−23.3 (−24.5 to −22.2)	30.1 (29.7 to 30.5)	−19.6 (−20.8 to −18.5)	35.5 (35.0 to 36.1)	−21.6 (−23.4 to −19.9)
25,000–<50,000	25.1 (24.8 to 25.4)	−6.1 (−7.2 to −5.1)	24.2 (23.9 to 24.6)	−6.8 (−7.8 to −5.7)	29.5 (29.0 to 30.1)	0.1 (−1.6 to 1.8)
50,000–<75,000	14.8 (14.6 to 15.0)	3.1 (2.3 to 3.9)	14.6 (14.4 to 14.9)	2.1 (1.3 to 2.9)	15.8 (15.4 to 16.3)	6.0 (4.8 to 7.2)
≥75,000	29.1 (28.8 to 29.4)	26.4 (25.4 to 27.4)	31.1 (30.7 to 31.4)	24.3 (23.2 to 25.3)	19.1 (18.7 to 19.6)	15.6 (14.1 to 17.0)
**No leisure-time physical activity^h^**	26.6 (26.3 to 26.8)	−9.4 (−10.5 to −9.4)	25.7 (25.4 to 26.0)	-6.9 (−7.9 to −5.8)	31.3 (30.8 to 31.9)	−8.4 (−10.1 to −6.7)
**Poor health^i^**	18.3 (18.1 to 18.6)	−11.7 (−12.6 to −10.8)	17.7 (17.4 to 18.0)	−9.4 (−10.3 to −8.5)	21.5 (21.1 to 22.0)	−12.1 (−13.5 to −10.7)
**Overweight or obese^j^**	64.5 (64.2 to 64.8)	−9.9 (−11.0 to −8.9)	63.7 (63.3 to 64.0)	−7.8 (−9.0 to −6.7)	68.7 (68.1 to 69.2)	−6.8 (−8.5 to −5.2)
**Hypertension^k^**	33.6 (33.3 to 33.8)	−8.7 (−9.7 to −7.6)	32.6 (32.3 to 33.0)	−6.2 (−7.2 to −5.1)	38.1 (37.6 to 38.7)	−10.1 (−11.7 to −8.5)
**High cholesterol^k^**	39.4 (39.1 to 39.7)	−5.0 (−6.2 to −3.8)	38.8 (38.4 to 39.2)	−3.5 (−4.6 to −2.3)	42.4 (41.8 to 43.0)	−4.8 (−6.6 to −3.1)
**Heart disease^k^**	6.9 (6.7 to 7.0)	−3.0 (−3.5 to −2.5)	6.5 (6.4 to 6.7)	−2.4 (−2.9 to −1.9)	8.6 (8.3 to 8.9)	−2.8 (−3.6 to −2.0)
**Arthritis^k^**	26.1 (25.9 to 26.4)	−7.0 (−7.9 to −6.1)	25.1 (24.9 to 25.4)	−5.2 (−6.1 to −4.3)	31.1 (30.6 to 31.6)	−6.8 (−8.3 to −5.4)
**Diabetes^k^**	10.6 (10.5 to 10.8)	−4.2 (−4.9 to −3.6)	10.4 (10.2 to 10.6)	−3.7 (−4.4 to −3.0)	12.0 (11.7 to 12.3)	−5.0 (−5.9 to −4.0)
**Current cigarette smoker^l^**	18.3 (18.1 to 18.6)	−7.8 (−8.7 to −6.9)	17.5 (17.3 to 17.8)	−5.8 (−6.7 to −4.9)	22.3 (21.8 to 22.8)	−7.4 (−8.8 to −5.9)
**Depressive disorder^k^**	18.0 (17.7 to 18.2)	−3.5 (−4.4 to −2.7)	17.5 (17.3 to 17.8)	−1.7 (−2.5 to −0.9)	20.1 (19.6 to 20.5)	−2.9 (−4.1 to −1.6)
**Asthma^k^**	14.0 (13.8 to 14.2)	−1.7 (−2.5 to −0.9)	14.0 (13.8 to 14.3)	−1.6 (−2.4 to −0.8)	13.9 (13.5 to 14.3)	−2.4 (−3.6 to −1.3)
**Chronic obstructive pulmonary disease^k^**	6.7 (6.6 to 6.8)	−4.0 (−4.5 to −3.5)	6.3 (6.2 to 6.5)	−3.3 (−3.8 to −2.8)	8.7 (8.4 to 9.0)	−4.3 (−5.2 to −3.5)

Abbreviations: —, not applicable; CI, confidence interval; metro, metropolitan; nonmetro, nonmetropolitan.

a Metropolitan and nonmetropolitan categories were created by using the Office of Management and Budget’s February 2013 delineations; US Census Bureau, Population Division; http://www.census.gov/population/metro/.

b County economic status was derived by using 2013 unemployment rate, per capita market income, and poverty rate for each county. An index was used to order counties into quintiles (poorest, poor, middle, affluent, and most affluent).

c Analyses excluded data from respondents in Alaska and Hawaii.

d Weighted percentages or percentage points and 95% CI.

e Unweighted sample sizes.

f Other race includes American Indian, Alaskan Native, Asian, Native Hawaiian, Other Pacific Islander, and multiracial.

g Reported for respondents aged 25 years or older.

h Self-reported no leisure-time physical activity within the previous month.

i Self-reported general health as fair or poor.

j Self-reported body mass index greater than 25 kg/m^2^.

k Self-reported being told they had the condition or disease. By definition, this excludes undiagnosed conditions or diseases.

l Self-reported smoking 100 cigarettes in their lifetimes and currently smoke cigarettes. By definition, this excludes former smokers.

We found differences in chronic disease and risk factors by economic status (Table 1). Large percentage-point differences between the most affluent and poorest counties were in 3 health outcomes and 3 risk factors: poor health (−11.7), hypertension (−8.7), arthritis (−7.0), body mass index classified as overweight or obese (−9.9), no leisure-time physical activity (−9.4), and current smoking (−7.8). All these differences were significant, with the poorest counties having the worst outcomes.

Estimates of chronic disease and risk factors also differed by metropolitan classification. Respondents in nonmetropolitan counties were significantly more likely to report chronic diseases (excluding asthma) and risk factors than were those in metropolitan counties. Differences between the most affluent and the poorest counties also varied by metropolitan classification. The largest percentage-point differences between metropolitan and nonmetropolitan counties were found for hypertension (metropolitan, −6.2; nonmetropolitan, −10.1), poor health (metropolitan, −9.4; nonmetropolitan, −12.1), arthritis (metropolitan, −5.2; nonmetropolitan, −6.8), current smoking (metropolitan, −5.8; nonmetropolitan, −7.4), and body mass index classified as overweight or obese (metropolitan, −7.8; nonmetropolitan, −6.8).

Hypertension was the disease for which the differences in estimates for each economic county group by metropolitan classification were largest. The prevalence of hypertension declined as economic county group improved for both metropolitan and nonmetropolitan counties; however, the difference was greater for nonmetropolitan counties (Figure). At every level of county economic status, metropolitan counties had a lower prevalence of hypertension than did nonmetropolitan counties.

**Figure:**
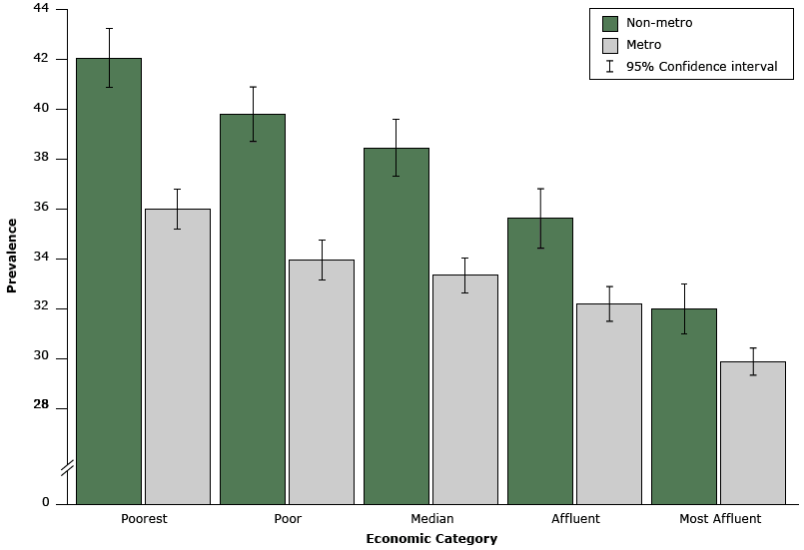
Prevalence (weighted estimates and 95% CIs) of hypertension by county metropolitan classification and economic category, adults (≥18 y), Behavioral Risk Factor Surveillance System, 2013. Hypertension was defined by self-report of ever having been told by a health professional that they had hypertension. Metropolitan and nonmetropolitan categories were determined by using the Office of Management and Budget’s February 2013 delineations and data from the U.S. Census Bureau, Population Division (http://www.census.gov/population/metro/). County economic status was determined by using 2013 unemployment rate, per capita market income, and poverty rate for each county. An index was used to order counties into quintiles (poorest, poor, median, affluent, and most affluent). Abbreviations: CI, confidence interval; metro, metropolitan; nonmetro, nonmetropolitan. **Table:** 

Economic Category	Metro (95% CI)	Nonmetro (95% CI)
Poorest	35.9 (35.1–36.8)	42.0 (40.8–43.2)
Poor	33.9 (33.1–34.7)	39.7 (38.6–40.8)
Median	33.2 (32.6–33.9)	38.4 (37.3–39.5)
Affluent	32.1 (31.4–32.9)	35.6 (34.4–36.7)
Most affluent	29.8 (29.2–30.3)	31.9 (30.9–32.9)

The prevalence of hypertension, arthritis, and poor health was lower in more affluent counties than in poor counties, and nonmetropolitan counties had higher rates than did metropolitan counties (Table 2). The prevalence of these conditions was higher or highest among those in the older age groups. Men reported hypertension more often than women, whereas women had higher rates of arthritis and poor health. Overall, among the 4 racial/ethnic groups studied, the highest prevalence of hypertension (42.8%) was among non-Hispanic blacks, the highest prevalence of arthritis (29.6%) was among non-Hispanic whites, and the highest prevalence of poor health (26.0%) was among Hispanics. The prevalence of each condition had an inverse linear relationship with education and household income (ie, the prevalence of each condition was lowest for those with the highest levels of education and income). The prevalence of hypertension and arthritis was higher among respondents with health insurance than among those without health insurance; those without health insurance had higher prevalence of poor health than those with health insurance. Hypertension, arthritis, and poor health were reported more often by respondents who were overweight or obese and by those who did not participate in physical activity. Current smokers reported arthritis and poor health more often than did nonsmokers, but hypertension prevalence did not significantly differ by smoking status.

**Table 2:** Prevalence of Hypertension, Arthritis, and Poor Health by County Economic Status^a^ and Metropolitan Classification^b^: Demographic Characteristics and Risk Factors of Adults Aged 18 Years or Older, Behavioral Risk Factor Surveillance System, 2013^c^

Characteristic	Hypertension^d^, % (95% CI)^e^	Arthritis^f^, % (95% CI)^e^	Poor Health^g^, % (95% CI)^e^
**County of residence**			
Poorest	38.6 (37.7–39.4)	29.7 (28.9–30.5)	25.5 (24.7–26.4)
Poor	37.0 (36.4–37.7)	30.0 (29.4–30.6)	21.8 (21.2–22.4)
Median	34.1 (33.4–34.7)	26.4 (25.9–27.0)	19.3 (18.8–19.8)
Affluent	32.5 (31.9–33.0)	25.4 (24.9–25.9)	16.7 (16.2–17.2)
Most affluent	29.9 (29.4–30.4)	22.7 (22.3–23.2)	13.9 (13.5–14.3)
**County metropolitan**			
Metropolitan	32.6 (32.3–33.0)	25.1 (24.9–25.4)	17.7 (17.4–18.0)
Nonmetropolitan	38.1 (37.6–38.7)	31.1 (30.6–31.6)	21.5 (21.1–22.0)
**Age, y**			
18–44	14.3 (13.9–14.6)	8.9 (8.6–9.2)	12.2 (11.9–12.6)
45–64	41.3 (40.8–41.8)	33.1 (32.6–33.5)	21.9 (21.5–22.3)
≥65	63.2 (62.7–63.7)	53.0 (52.4–53.5)	25.9 (25.4–26.4)
**Sex**			
Male	35.1 (34.7–35.6)	22.0 (21.7–22.4)	17.4 (17.1–17.8)
Female	32.1 (31.7–32.5)	29.9 (29.6–30.3)	19.1 (18.8–19.5)
**Race/ethnicity**			
Hispanic	24.8 (23.9–25.8)	14.4 (13.7–15.1)	26.0 (25.1–27.0)
White, non-Hispanic	34.6 (34.3–34.9)	29.6 (29.4–29.9)	15.9 (15.7–16.2)
Black, non-Hispanic	42.8 (41.8–43.8)	25.8 (25.0–26.6)	23.1 (22.3–23.9)
Other, non-Hispanic **h**	27.2 (25.8–28.7)	19.3 (18.1–20.5)	15.5 (14.4–16.6)
**Education^i^**			
<High school graduate	43.6 (42.5–44.6)	34.2 (33.2–35.2)	41.6 (40.5–42.6)
High school graduate	41.5 (40.9–42.0)	32.9 (32.4–33.4)	22.3 (21.9–22.8)
Some college	37.4 (36.9–38.0)	30.6 (30.1–31.1)	16.9 (16.4–17.3)
College graduate	28.9 (28.5–29.4)	21.6 (21.3–22.0)	7.9 (7.6–8.1)
**Annual household income, $**			
<25,000	38.6 (38.0–39.2)	31.6 (31.1–32.2)	33.4 (32.8–34.0)
25,000–<50,000	35.8 (35.2–36.4)	27.5 (27.0–28.1)	17.5 (17.0–18.1)
50,000–75,000	32.5 (31.8–33.2)	24.7 (24.0–25.3)	10.4 (9.9–10.9)
≥75,000	26.8 (26.3–27.3)	19.3 (18.9–19.7)	5.9 (5.7–6.2)
**Health insurance^j^**			
Yes	35.7 (35.4–36.0)	28.4 (28.2–28.7)	17.3 (17.0–17.5)
No	23.7 (23.0–24.4)	15.3 (14.8–15.9)	23.6 (22.9–24.3)
**Overweight or obese^k^**			
Yes	41.0 (40.7–41.4)	30.1 (29.8–30.4)	20.9 (20.6–21.2)
No	20.6 (20.2–21.0)	19.6 (19.3–20.0)	13.7 (13.3–14.1)
**Current cigarette smoker^l^**			
Yes	32.8 (32.2–33.5)	28.7 (28.1–29.3)	26.8 (26.1–27.4)
No	33.9 (33.5–34.2)	25.8 (25.5–26.1)	16.4 (16.1–16.6)
**Physical activity^m^**			
Yes	30.8 (30.5–31.2)	23.7 (23.4–24.0)	13.7 (13.4–13.9)
No	42.1 (41.5–42.7)	34.5 (34.0–35.1)	30.7 (30.2–31.3)

Abbreviation: CI, confidence interval.

a County economic status was determined by using 2013 unemployment rate, per capita market income, and poverty rate for each county. An index was used to order counties into quintiles (poorest, poor, median, affluent, and most affluent).

b Metropolitan and nonmetropolitan categories were created by using the Office of Management and Budget’s February 2013 delineations; US Census Bureau, Population Division; http://www.census.gov/population/metro/.

c Analyses excluded data from respondents in Alaska and Hawaii.

d Self-reported being told by a health professional that they had high blood pressure.

e Weighted percentage and 95% confidence interval.

f Self-reported being told by a health professional that they had some form of arthritis, rheumatoid arthritis, gout, lupus, or fibromyalgia.

g Self-reported general health as poor or fair.

h Other race includes American Indian, Alaskan Native, Asian, Native Hawaiian, Other Pacific Islander, and multiracial.

i Reported for respondents aged 25 years or older.

j Reported having health insurance at the time of the survey.

k Self-reported height and weight equivalent to body mass index greater than 25 kg/m^2^.

l Self-reported smoking 100 cigarettes and currently smoke cigarettes. By definition, this excludes former cigarette smokers.

m Self-reported leisure-time physical activity within the previous month.

After we adjusted for covariates, an association between county economic status and hypertension, arthritis, and poor health remained (Table 3). The prevalence of hypertension in the poorest counties was 9% higher than in the most affluent counties (adjusted prevalence ratio, 1.09; 95% CI, 1.05–1.12). For arthritis, the poorest counties had 13% higher prevalence than the most affluent counties (adjusted prevalence ratio, 1.13; 95% CI, 1.09–1.18). The greatest difference in prevalence rates among the 3 conditions was found for poor health, with the poorest counties having 15% higher prevalence than the most affluent counties (adjusted prevalence ratio, 1.15; 95% CI, 1.10–1.21).

**Table 3:** Adjusted Prevalence Ratios of Hypertension, Arthritis, and Poor Health, Adults Aged 18 Years or Older, Behavioral Risk Factor Surveillance System, 2013^a^

Characteristic	Hypertension^b^, APR (95% CI)^e^	Arthritis^c^, APR (95% CI)^e^	Poor Health^d^, APR (95% CI)^e^
**County of residence^f^**			
Poorest	1.09 (1.05–1.12)	1.13 (1.09–1.18)	1.15 (1.10–1.21)
Poor	1.06 (1.04–1.09)	1.11 (1.08–1.14)	1.10 (1.05–1.14)
Median	1.04 (1.02–1.07)	1.06 (1.03–1.09)	1.07 (1.02–1.12)
Affluent	1.03 (1.01–1.06)	1.06 (1.03–1.08)	1.03 (0.99–1.07)
Most affluent	1 [Reference]		
**County metropolitan^g^**			
Metropolitan	1 [Reference]		
Nonmetropolitan	1.03 (1.01–1.04)	0.99 (0.97–1.02)	0.99 (0.96–1.02)
**Age, y**			
18–44	1 [Reference]		
45–64	2.23 (2.16–2.30)	2.65 (2.56–2.75)	1.67 (1.60–1.74)
≥65	3.36 (3.26–3.47)	3.87 (3.73–4.02)	1.72 (1.64–1.80)
**Sex**			
Male	1 [Reference]		
Female	0.90 (0.88–0.91)	1.28 (1.25–1.30)	1.02 (0.99–1.05)
**Race/ethnicity**			
Hispanic	0.90 (0.86–0.93)	0.63 (0.60–0.67)	1.24 (1.18–1.30)
White, non-Hispanic	1 [Reference]		
Black, non-Hispanic	1.28 (1.24–1.31)	0.86 (0.83–0.89)	1.09 (1.04–1.14)
Other, non-Hispanic^h^	1.09 (1.04–1.14)	0.93 (0.88–0.98)	1.16 (1.07–1.25)
**Education^i^**			
<High school graduate	1.14 (1.10–1.18)	1.23 (1.18–1.28)	1.91 (1.81–2.02)
High school graduate	1.10 (1.07–1.12)	1.11 (1.08–1.13)	1.33 (1.27–1.39)
Some college	1.10 (1.08–1.12)	1.17 (1.14–1.20)	1.29 (1.23–1.35)
College graduate	1 [Reference]		
**Annual household income, $**			
<25,000	1.32 (1.29–1.36)	1.55 (1.50–1.60)	3.63 (3.41–3.86)
25,000–<50,000	1.15 (1.12–1.18)	1.24 (1.20–1.28)	2.10 (1.97–2.23)
50,000–<75,000	1.09 (1.06–1.12)	1.13 (1.10–1.17)	1.43 (1.33–1.53)
≥75,000	1 [Reference]		
**Health insurance^j^**			
Yes	1 [Reference]		
No	0.84 (0.82–0.87)	0.73 (0.70–0.76)	0.86 (0.83–0.91)
**Overweight or obese^k^**			
Yes	1.68 (1.64–1.72)	1.37 (1.34–1.41)	1.28 (1.23–1.32)
No	1 [Reference]		
**Current cigarette smoker^l^**			
Yes	1.05 (1.02–1.07)	1.19 (1.16–1.22)	1.31 (1.27–1.35)
No	1 [Reference]		
**Physical activity^m^**			
Yes	1 [Reference]		
No	1.11 (1.09–1.14)	1.15 (1.13–1.18)	1.58 (1.54–1.63)

Abbreviations: APR, adjusted prevalence ratio; CI, confidence interval.

a Analyses excluded data from respondents in Alaska and Hawaii.

b Self-reported ever being told by a health professional that they had high blood pressure.

c Self-reported ever being told by a health professional that they had some form of arthritis, rheumatoid arthritis, gout, lupus, or fibromyalgia.

d Self-reported general health as poor or fair.

e Adjusted prevalence ratio and 95% confidence interval; multivariable logistic regression model included all variables in the table.

f County economic status was created by using 2013 unemployment rate, per capita market income, and poverty rate for each county. An index was used to order counties into quintiles (poorest, poor, median, affluent, and most affluent).

g Metropolitan and nonmetropolitan categories were created by using the Office of Management and Budget’s February 2013 delineations; US Census Bureau, Population Division; http://www.census.gov/population/metro/.

h Other race includes American Indian, Alaskan Native, Asian, Native Hawaiian, Other Pacific Islander, and multiracial.

i Reported for respondents aged 25 years or older.

j Reported having health insurance at the time of the survey.

k Self-reported height and weight equivalent to body mass index greater than 25 kg/m^2^.

l Self-reported smoking 100 cigarettes and currently smokes cigarettes. By definition, this excludes former smokers.

m Self-reported leisure-time physical activity within the previous month.

## Discussion

We examined the association between county economic status and prevalence of chronic disease and associated risk factors. For hypertension, arthritis, and poor health, the prevalence was lower as county economics improved. This association remained after we adjusted for covariates. To our knowledge, this is the first study to examine national prevalence of chronic disease based on county economics using a rigorous economic index.

Our results are consistent with those of previous research on socioeconomic status and health outcomes. Increased mortality and poor health is associated with area-level poverty even after the data are adjusted for individual risk factors (16,17). Research also shows an increase in heart disease among residents in disadvantaged neighborhoods (18). Limited research has been conducted on arthritis and area-level poverty in the United States, although a North Carolina study showed an interaction effect between community poverty and low individual socioeconomic status (19).

Several characteristics related to the gradient in health and economic status are potentially modifiable. These include access to health care, dissemination of evidence-based community-delivered public health interventions, and environmental barriers to healthy food and physical activity. Residents of poor counties might have more barriers to accessing health care services than those in affluent counties. Increased access to services, such as Medicaid, is likely to benefit poor counties the most, since more residents in these counties than in affluent counties would qualify for these services.

Our findings also suggest that the higher prevalence of hypertension, arthritis, and poor health in nonaffluent counties makes these areas a prime target for clinical–community linkages in the form of greater dissemination of evidence-based, community-delivered, self-management education and physical activity programs. For example, a recent meta-analysis of the Chronic Disease Self-Management Program showed that this program can generate sustained (9–12 months) improvements in several measures of psychological health (eg, depression, health distress, self-efficacy), aerobic exercise, and cognitive symptom management and at least 4-to-6–month improvements in energy, fatigue, and self-rated health (20). The benefits of evidence-based public health interventions suggest that these programs may be especially meaningful and important components in a comprehensive strategy to improve health outcomes and in the primary and tertiary prevention of chronic disease in economically disadvantaged US counties.

The food environment in poor counties may also contribute to their higher prevalence of chronic diseases. Density of fast-food restaurants and convenience stores tends to be higher in poor neighborhoods than in affluent neighborhoods (21). The number of fast-food restaurants and convenience stores is positively associated with mortality and diabetes (22). Methods for bringing healthy food into poor counties include neighborhood farmers markets, community-supported agriculture, cooperative grocery stores, community gardens, and mobile stores (23). Interventions aimed at increasing access to healthy food might improve the health of residents in poor counties.

A third factor that might affect chronic disease prevalence in poor counties is the physical environment. Neighborhoods with low socioeconomic status are less likely to have access to parks and recreation facilities or to have an environment that supports active transportation (eg, walking or biking to work), less likely to be close to commercial areas, schools, and work, and less likely to have safe walkable routes (24) to any place. Establishing programs and implementing policies that promote safe routes to school and work and community bicycling increase active transportation (25). Improving built-environment characteristics is associated with increased physical activity and could improve the health of residents in poor counties.

This study has several limitations. First, BRFSS data are subject to nonreporting and social desirability bias. Second, biases might differ by geographic location, as was found for obesity (26). Third, interviews were limited to respondents living in noninstitutionalized settings, which excludes residents of skilled nursing facilities and retirement homes, and might result in an underestimation of the chronic disease burden. Finally, county of residence was determined according to the current residence of the respondent, which did not account for previous places of residence.

Our study also has important strengths. We extend the literature on associations between local economic activity for 2 important chronic conditions, hypertension and arthritis, and one important representation of health status, self-rated health. Hypertension and arthritis are 2 of the most common chronic conditions affecting US adults (27). Additionally, self-rated health is a robust global measure of health and has associations with morbidity and mortality (28,29); self-rated health is also sensitive to changes in neighborhood and individual-level social capital (30). By establishing a relationship between hypertension, arthritis, and poor health with county-level economics, we identified meaningful target groups by condition and by geographic area in which gains in public health might occur with appropriate intervention. Other strengths of our study include using a population-based data source with sufficient sample size to find small to modest adjusted associations and using an accepted (used by ARC) economic index with enough levels to illustrate meaningful variability at the county level.

Residents in poor counties have a greater prevalence of hypertension, arthritis, and poor health than residents in affluent counties. Furthermore, while a social gradient exists, with increases in poor health outcomes as county-level economic status decreases, we have explicitly documented this association for 2 highly prevalent chronic conditions and poor self-rated health for virtually all counties in the contiguous United States. Improvements to food and built environments and access to health care services and evidence-based, community-delivered public health interventions in counties with an economic status below that of the most affluent might help decrease the prevalence and impact of chronic disease overall and decrease the disparities seen in poor counties.
